# Perceptions on Connecting Respite Care Volunteers and Caregivers

**DOI:** 10.3390/ijerph17082911

**Published:** 2020-04-23

**Authors:** Solange Campos-Romero, Valeria Herskovic, Carolina Fuentes, Esmeralda Abarca

**Affiliations:** 1 School of Nursing, Pontificia Universidad Católica de Chile, Santiago 8940000, Chile; scamposr@uc.cl (S.C.-R.); eaabarca@uc.cl (E.A.); 2 Department of Computer Science, Pontificia Universidad Católica de Chile, Santiago 8940000, Chile; 3 School of Computer Science, University of Nottingham, Nottingham NG8 1BB, UK; Carolina.Fuentes@nottingham.ac.uk; 4 School of Computer Science & Informatics, Cardiff University, Cardiff CF24 3AA, Wales, UK

**Keywords:** caregiving, IT, respite, volunteer, qualitative research

## Abstract

The most common requirement for informal caregivers is to experience a respite or temporary break from their caregiving routine. Some initiatives have been undertaken to provide respite care through volunteer providers. We report on a qualitative study carried out in Santiago, Chile, to learn about the willingness of potential volunteers to provide respite care for bedridden older persons, as well as their willingness to use information and communication technologies (ICT) to connect to caregivers in a low-income neighbourhood within their own geographic district. A trustworthy institution that mediates the volunteer–caregiver relationship is considered to be important by potential volunteers. Potential volunteers were found to be willing to use ICT to provide respite care, sharing basic information about themselves. However, they were also aware of the digital skill gap that may exist between them and the caregivers and were distrustful of unknown websites that could connect them to care recipients.

## 1. Introduction

Functional dependence is typically measured as the ability of older persons to perform instrumental activities of daily living, e.g., bathing and shopping [1]. Dependent older persons are usually cared for by informal caregivers, i.e., usually unpaid family members and close friends. This role is a major life stressor and can impact the health and well-being of such caregivers [2]. 

The most common requirement for caregivers is to experience a respite, or temporary break from their caregiving routine [3]. As such, respite care has been found to help reduce depression, burden and anger among caregivers [4]. 

Volunteers are people who are willing to give their time to help another person, group or organization [5], and a number of initiatives have been undertaken to provide respite care through the use of volunteers [6]. From the perspective of volunteers, training programmes have been found to be a relevant factor in improving caregiving outcomes [7]. However, in one particular volunteer caregiving study, only 58% of volunteers were willing to provide respite services after having received training [8]. Volunteering is linked to the well-being of the older person providing the volunteering [9], becoming a protective factor for the negative effects of low self-esteem [10]. Particularly, individuals who volunteer as respite caregivers tend to be emotionally resilient [11]. 

Informal caregivers have been found to have a favourable outlook towards accessing respite assistance through technology, showing a willingness to share basic information about themselves, despite valuing the importance of face-to-face contact [12]. Accordingly, the technology that is deployed should correspond to a peer-to-peer system to help link caregivers with willing volunteers. However, in order to use such a system, certain digital skills are necessary. This is important because in Chile, 69% of 55-to-65 year olds lack experience in the use of technology, compared to only 9% of 16-to-29 year olds [13]. Although caregivers show a willingness to initiate contact with volunteers through technological means, research into how volunteers feel about this is limited. Therefore, this study aims to understand the willingness of potential volunteers (people who are not currently volunteering to provide respite care but are interested in doing so) of respite care for bedridden older persons, as well as their willingness to use technologies, which, if deployed, would enable the provision of such services to a low-income and underserved segment of society in Santiago, Chile. First, we provide some context about the Chilean health care system and the situation of older adults and their caregivers in Chile today.

## 2. Context

Chile is ageing, at an even faster rate than many other countries, due to improved living conditions and a higher life expectancy [14]. While currently those over 60 are around 15.7% of the population, it is expected that by 2050, over 32.9% of people in Chile will be older than 60, and 10.3% will be older than 80 [14].

Chile is a country with high inequality, stemming from two sources: differences between individuals, and geographical differences [15]. Peñalolén is a neighbourhood, or district, within Santiago, the capital city of Chile (population: 6.7 million), that encompasses all socioeconomic groups, although they are highly segregated within the district, i.e., some areas concentrate high income residents, while others are populated with low income residents [16]. 

The Chilean healthcare system incorporates both public and private healthcare providers. Over 76.5% of the population in urban areas are users of the public system [17]. These users can choose to pay to see a healthcare provider of their choice or attend an assigned healthcare centre. 

The National Service for Older Adults, SENAMA (Servicio Nacional del Adulto Mayor) was created in 2002 to improve conditions for older people across the country [18]. Among its current programmes, one is aimed at dependent older adults with no main caregivers, while another allows older adults to attend a day programme in a facility, of which there are 16 in Santiago and none in Peñalolén [19]. When it comes to caregiving support, formal governmental support is weak in Chile, making the families mainly responsible for providing caregiving [20,21]. Although some government programmes have been created to help caregivers (e.g., *Chile Cuida*), coverage is extremely low (it only operates in two—out of 346—districts) [20].

Some local district governments (*municipalidad*, in Chile) have put in place programmes to support dependent older adults at home, providing low complexity instrumental care of older adults, e.g., companionship care, feeding and recreational activities, among others. One such district, Las Condes, which is generally a high income area, has a volunteering programme which started in 2003 and supports fragile older adults by connecting them with healthy adults over 50 [22]. In this case, the volunteers share three hours of their time with the older adults, providing them companionship, reading, conversation and walking.

The nongovernmental organization (NGO) *Hogar de Cristo* is a national organization that provides support to older adults. They have several centres where older adults live, and they also have a domiciliary program for older adults, which supports older adults and their caregivers at home through professionals, volunteers and student interns. Volunteers and interns provide psycho-affective companionship support both to caregivers and older adults [23]. Some local NGOs also have created similar programmes, e.g., *Amanoz* in Peñalolén, which has a small pilot intervention of seven caregivers of older adults with dementia and seven volunteers, who are supported by a psychologist and a nurse technician [24]. 

All the previously discussed initiatives are not enough to cover the need for caregiving support and are not unified; some are sporadic interventions and they follow different guidelines for integrating volunteers and the kind of support provided. In Chile, as in many other societies, the care of older adults is usually the responsibility of a close family member, with generally strong affective links [25]. Only about 15% of older adults live on their own [18]. Still, the caregiving burden overwhelmingly falls to women in Chile [26]. Community support for these women is practically non-existent, and furthermore, in situations of poverty, lack of resources and training, and lack of a respite, caregivers face increased risks to their health and well-being [18].

Although Chile has a higher number of social civil organisations than other countries, such as Australia and the U.S., only around 13% of people participate in some volunteer activity [27]. Still, Chile is among the Latin American countries with higher volunteer participation—one study found that 91% of third sector institutions (NGOs, foundations, corporations) rely on volunteer work to operate [28]. SENAMA has identified 11 NGOs that support 31,960 older adults—however, it is not clear how many of these initiatives support caregivers, nor how many use volunteers. Furthermore, the current population of older adults in Chile is over three million [29], so there is a lack of policies and initiatives to help this population [14].

## 3. Materials and Methods

An exploratory, descriptive and comprehensive study was undertaken using a qualitative methodology to investigate the acceptance of information and communication technologies among potential volunteer providers of respite care. This study is part of a larger project in understanding how to connect volunteers and caregivers in an underserved segment of population in Peñalolén, Chile. Within this project, we had previously conducted a related study from the caregivers’ point of view, analysing their willingness to use technology to connect with volunteers [12]. We found that caregivers had a favourable disposition to connect with volunteers using a peer-to-peer economy-based system. In this new study, we explore this same issue but from the perspective of the volunteers. Therefore, for this study, we took the questions from the previous study aimed at the caregivers [12] and adapted them for the potential volunteers. The questions can be found in Appendix A. Questions 1–4 pertain to general contextual information, used to better understand each participant, while questions 5 and 6 are very close to the goal of this study and therefore the answers to these questions were used to obtain our results.

### 3.1. Participants

Potential volunteers belonging to a religious community were invited to participate, in line with the following inclusion criteria: over 18 years of age, with the ability to read and write. The exclusion criterion included the presence of any type of disability or functional limitation, such as deafness or blindness.

The group of participants consisted of 8 people, all women. The average age was 49.1 (min 23, max 65), with an average education period of 13.8 years (min 2, max 17), with 7 participants having completed higher education. Regarding participant occupations, there were 2 housewives, a housewife-podiatrist, a stylist, an industrial engineer, a special needs educator, a chef who worked for her own company, and a direct sales executive. All volunteers had previous experience as caregivers, mainly taking care of dependent older adults and also taking care of other members from the religious community. We did not explore in detail the volunteers’ past experiences as caregivers.

The digital competence of the volunteers was evaluated using the Digital Competence framework [30], which categorizes people into one of the following four categories of digital skills: “none,” “low,” “basic” and “above basic.” The results indicated that the majority of the volunteers possess a certain degree of digital competence. Furthermore, 7 out of the 8 participants were smartphone users. Regarding online access, all participants reported having an internet connection from home.

Participants were contacted via a request submitted to a Church pastor, who subsequently authorized the cooperation of his religious community with the research. This particular religious community carries out regular volunteer initiatives, although volunteering aimed at bedridden individuals is uncommon. Therefore, the participants of this study are deemed *potential* volunteers—people who would be interested in volunteering to provide caregivers of bedridden older individuals a respite. A member of this religious community, who was selected by the Pastor to reach out to the congregation, was responsible for compiling a list of telephone numbers of people interested in participating in the study. Subsequently, one of the researchers established contact with the volunteers via telephone and explained the purpose and methodology of the research. The individuals who accepted to participate were invited to attend an introductory appointment in which they were formally registered as part of the research via an informed consent process. Compensation of 10,000 Chilean pesos (15 U.S. dollars) was provided to participants.

### 3.2. Data Collection

In-depth, individual semi-structured interviews (using the questions in Appendix A) were carried out by one researcher in participant homes or on Church property, as per the request of each volunteer. Each interview lasted between 45 and 60 min. All interviews were recorded and transcribed verbatim.

### 3.3. Data Analysis

A descriptive qualitative analysis of the interview data was undertaken by conducting content analysis using the open coding methodology pertaining to Grounded Theory [31,32]. The analysis process was carried out by two researchers, experts in qualitative analysis, as follows. Before beginning the analysis, the researchers manifested their previous personal and professional experiences with the topic of study in order to identify any possible influences in the analysis. After the interviews, data was transcribed by another person (not either of the researchers participating in the analysis). Then, for each interview, each researcher read the transcript, asking the following questions for each paragraph: *What is this fragment talking about?* and *What does it say about that topic?* The answers to these questions generated concepts that began forming codes, which were constantly compared with other codes to identify similarities and differences between them. Then, they were grouped under more abstract terms to generate categories and subcategories. To ensure methodological rigour in the quality of the results, we used peer debriefing to reduce personal biases, i.e., each researcher carried out this process independently, then in conjunction with the other researcher to resolve any discrepancies until consensus was reached and an intersubjective agreement was formed with regard to the categories generated.

The previous process was repeated for each interview until data saturation was reached, i.e., no new codes or categories were generated. This happened at the sixth interview, which was confirmed by two subsequent interviews, thereby ensuring an overall sample of eight participants. Result feedback was subsequently provided to two of the participants, who confirmed that the findings faithfully represented their sentiments.

In total, our analysis generated five categories; three of which pertained to the aim of this study (corresponding to questions 5 and 6), so we discuss these three categories in Section 4.

### 3.4. Ethical Considerations

The project was approved by the Scientific Ethics Committee of the Pontificia Universidad Católica de Chile (Reference Number: 15-334). All participants agreed to take part in line with the informed consent process.

## 4. Results

Three main categories emerged from the descriptive analysis of the eight interviews conducted with the volunteers: “institution responsible for volunteering,” “communication channels between volunteer and caregiver” and “background to be shared through information technology.” These categories are outlined in detail below.

### 4.1. Institution Responsible for Volunteering

#### 4.1.1. Characteristics of the Volunteering Institution

Volunteers believe that volunteering should be carried out via a structured, sound and recognised institution, such as a municipality, which should be made up of responsible individuals. Results identified that the existence of this institution would generate trust and security among volunteers in order to help older persons and favour caregiver respite.

*“The trust comes from us being under your protection, that is to say, of the research project [...]. It is not the same thing if you go somewhere alone to volunteer because, in that case, you won’t know anything, I mean, you’ll be totally rudderless. If you go to a place, to an institution you trust [...] you know where they came from and who is in charge. That gives you a kind of security...”* (V03: Female, 65, low digital competence).

#### 4.1.2. Roles of the Volunteering Institution

Participants identified a number of roles that they believed should be performed by the volunteering institution, which are described in this section. First, the institution should be the one to establish the link between volunteer, caregiver and older person, considering the characteristics of each.

*“The caregivers come together, you know, the two different sides, so you more or less bring together or match the two sides that have the characteristics required by each caregiver with the abilities of the volunteers.”* (V05: Female, 60, basic digital competence).

Second, the institution should be in charge of ensuring that volunteer safety and comfort are guaranteed.

*“I think it has to be a place, I mean, I don’t care if it’s modest or not, but it has to be a place where I feel safe. For example, if I’m taking care of someone and I suddenly hear gunfire or, I don’t know, their violent or drunk son or daughter is coming over... I would feel very uncomfortable.* (V01: Female, 28, above basic digital competence).

Third, the institution should also ensure that the volunteer undergoes the necessary training in medical or other care procedures to provide meaningful care for older persons.

*“I think that training on your part should include the issue of administering medication. It could include the care of the patient too, if they had, bandages, how to take care of those bandages, the hygienic part. If the bandages are to be left alone, for how long, whether the caregiver is going to clean them, or what to do in such cases [...] How to change them, how to bathe them if necessary.”* (V05: Female, 60, basic digital competence).

Finally, the volunteers not only want an initial screening process and some training in medical procedures, but also continuous monitoring by the institution. The volunteers view this process as an evaluation of their work by the care recipient, and a process in which they can provide feedback to the institution about their experience. That is, the volunteers expect this to be an ongoing process.

*“Not feeling alone after the initial connection and having a follow-up, you know, (the volunteer) going to the house, then leaving the house, and following that up with a “How did it go? Did you have any problems, any difficulties? Was everything alright? Do you need anything? Do you think you could visit again?” Keeping track of everything, as well as the questions from the person you went to see.”* (V04: Female, 56, above basic digital competence).

### 4.2. Communication Channels between Volunteer and Caregiver

Channels of communication between a volunteer and caregiver represent the different ways in which to identify potential volunteers who can be connected with informal caregivers who, in turn, can access the required respite. Volunteers report that technological devices could contribute to encounters with informal caregivers. This includes communication via either smartphones or regular telephones that have no internet functionality.

#### 4.2.1. Communication Via Smartphone

Communication between an informal caregiver and a volunteer may be established through the use of a smartphone, whereby either party is able to make telephone calls and send text messages or messages via an online application, such as WhatsApp. Any such online application must be easy to use, password-free, and offer the functionality to generate both voice and written messages.

*“Using a mobile phone, being able to say things to each other, like ‘hey, actually, this happened, this other thing happened,’ over the phone via messages or, I would communicate with WhatsApp, via audio messages, as well as WhatsApp calls, which I know how to use. Using the phone to communicate with the person’s family, with the caregiver.”* (V06: Female, 65, basic digital competence).

#### 4.2.2. Telephone-Only Communication

Certain volunteers prefer to establish communication with the informal caregiver via the channel of a traditional telephone. Reasons identified include that some older persons may not know how to use more modern types of information communication technology (ICT), while others may lack access to the internet. Furthermore, certain volunteers refer to a mistrust of certain websites, e.g.:

*“(…) But I don’t trust these internet things, like those sites where they find a romantic partner and things like that, no. I like things on paper, having the information and making the connection, more personal, in person.”* (V04: Female, 56, above basic digital competence).

Some volunteers give a higher importance to personal relationships, rather than those formed via ICTs (as evidenced in the next quote, as well as the previous one).

*“This ICT thing is very trendy and works well, for a certain percentage of the population, but it turns out that people need to use what works for them, in this case the caregivers and everything, the elderly... I think it largely depends on who the caregiver is [...]. I would have to know that ‘this person is over 40, let’s play it safe and call them (on the telephone).’”* (V08: Female, 23, above basic digital competence).

### 4.3. Personal and Background Information to be Shared through Information Technology

The personal and background information that is to be shared through a particular communications technology of all involved (volunteers, caregivers, and older adults) includes establishing the minimum information necessary about each party in order for the respite services to be undertaken, as well as what each party is willing to disclose. Accordingly, this includes information related to the background of the volunteer, the older person and the caregiver.

#### 4.3.1. Volunteer Information

Volunteers would provide basic personal information, such as name, age, occupation and marital status. They would provide information related to the areas in which they are able to assist the older person and the time they have available to do so.

*“I don’t think too much detail is needed. Just things like my age, my name, what I do, that I’m married, I don’t have children, I’m planning to have children, etc.”* (V01: Female, 28, basic digital competence).

Some volunteers thought they should provide much more information, e.g., via a personality report to the organization through which they are volunteering.

*“Well, who I am, my full name, my address, what I do, where I’m volunteering, if there is anyone who knows me. You know, like how you made contact with the lady from the church, the people who know who I am [...]. First of all, you have to find out about me as well. I can provide my personality report if needed, no problem at all.”* (V02: Female, 45, basic digital competence).

#### 4.3.2. Older Person Information

Volunteers would like to have access to as much information as possible about the older person, such as their name, age, health condition, how long they have been with their current health condition, their prognosis and the type of care required.

*“Everything. I want to know everything about the person I’m going to take care of. Everything, like name, how long they have been this way, age, what (condition) they have, what is their medium-term outlook, everything. And what I would have to do too, that would be ideal.”* (V01: Female, 28, above basic digital competence).

#### 4.3.3. Informal Caregiver Information

Volunteers would like to know the name, age and occupation of the caregiver, as well as their relationship with the older person, how much time they spend caring for them, and what recreational activities the caregiver performs with them, in order for the volunteer to be able to undertake similar activities with that person as necessary.

*“I would like to know their relationship with the patient, I would like to know how much time they spend with that person, what they talk about, the activities they carry out with the patient, because if the caregiver plays cards with the patient, I’ll know not to bring a word search puzzle, for example, so those kinds of things are important.”* (V05: Female, 60, basic digital competence).

## 5. Discussion

The volunteers who took part in our study were potential volunteers who would be able to contribute to support caregivers on their older adult care tasks. The results of this research show that potential volunteers are interested in the presence of a volunteering institution to help systematize and structure the respite provided for the informal caregiver and the assistance for the older person. In fact, relevant literature indicates that a volunteering institution (a non-profit organization), by means of its constituent volunteer coordinators and staff, is responsible for a number of aspects, including recruiting and training volunteers, identifying volunteer opportunities, conducting volunteer follow-up and acknowledgement activities, coordinating and planning volunteer work, compiling volunteer databases and generating progress reports, and leading the provision of emotional support, among others [33,34]. There is strong agreement on this issue between volunteers and caregivers, who request that volunteers are members of an institution that initiates contact between volunteers and caregivers [12]. In the case of our study, the relevance of having a main institution regulating volunteer roles could be part of the safety concerns of volunteers.

Potential volunteers who would perform organized work through a volunteering institution indicate that they would feel an obligation towards that entity in relation to fulfilling previously assumed schedules and duties. Simultaneously, they would feel that their volunteering efforts were generating a greater impact on the beneficiary person since the volunteering institution provides a rigid structure through which intentional activities are directed and fulfilled to the benefit of a specific target community [35].

The results show how volunteers deem that trust in the institution to safeguard the well-being and comfort of the volunteer is a relevant factor. This particular factor is likely to be exacerbated in this research study due to the fact it was undertaken in a geographic location which is home to a number of potentially unsafe neighbourhoods. Thus, belief in the institution that organizes volunteering is a key factor in terms of generating trust between volunteers and caregivers [36]. According to relevant literature, trust may be an opinion based on the belief that someone to whom we relate will act in the common interest and that neither party will attempt to harm the other by exploiting its respective weaknesses [37,38]. Indeed, trust has been identified as a key factor in the development of volunteering organisations in general [36]. Trust is an especially delicate issue in this type of system, in which deeply personal information may be shared through digital means.

According to the results of this study, potential volunteers are willing to use ICT to establish communication with caregivers and to ensure respite is achieved. In additional research [35,39], ICTs are used by volunteers, regardless of whether they are a member of a volunteering institution or not, to carry out volunteering and to activate the social structures involved therein. Importantly, ICT enables volunteers and the volunteering institution, if the former is a member of such an organization, to plan and coordinate systematic or periodical volunteering activities. A systematic activity relates to a repeated pattern of help provided over time and which favours the bringing together of new people and the development of relationships within a group [35,39].

A further result of this study relates to the ways in which potential volunteers communicate among themselves, with the organizing institution and with the beneficiaries of the respite. The findings show that this communication could be undertaken by means of existing ICTs and social networks, i.e., without the creation of new information systems or technologies. These results are similar to those found in literature [33,39] in which volunteers use telephone calls, text messages and messages sent via Facebook, Twitter, and/or WhatsApp to complete their volunteer activities, e.g., in the case of micro-coordination of volunteering work and fundraising duties. Conversely, the coordination, planning, discussion, reminder of and feedback on voluntary work on a larger scale is carried out through the use of emails and productive software, such as Google Docs spreadsheets, documents and forms [35,39,40,41]. Mobile telephones, laptops and other devices with an internet connection were used as resources to establish immediate communication. These ICTs provide, in differing ways, an infrastructure for volunteer work, i.e., an invisible support, essentially relational and embedded within the social structure of the participants in order to enable the development of a social network of collaborative work [35,42,43,44].

According to the results of this research, volunteers believe that ICTs act as a support for establishing communication with the caregiver, thereby emphasizing the importance of human relationships. In effect, social computing technologies are used to establish and contribute towards the success of bridge-building work [39] and social networks are used to create and maintain social capital [45].

Furthermore, in their research with young adult volunteers, Voida, Yao and Korn (2015) found that the social structure of volunteering, i.e., the nature of the relationship between the volunteer and the beneficiary, as well as the nature of the relationship between the volunteers themselves, determines how volunteer work is undertaken. On one hand, the closer the relationship between the volunteer and the beneficiary, the greater the likelihood that meaningful help will be provided. On the other hand, the closer the relationship between the volunteers themselves and the more they are guided by the same ideals and objectives, the more likely it is that help will be provided and that it will be enjoyable at the same time. In addition, the social structure would allow new volunteers to join a volunteering institution as a result of a close personal contact, such as a friend or family member, already being a member of that organization. For volunteer coordinators, interaction between themselves, volunteers and beneficiaries is the most relevant task undertaken during their work [39]. In fact, certain volunteer coordinators have expressed concern about the use of social computing technologies because they believe, by means of their use, they are unable to know for certain who is joining the volunteering activity or what their genuine motives are. Therefore, they would place a greater value on recruitment being undertaken by personal interviews [39].

There is evidence that a digital divide still persists, even in developed countries. However, this divide has evolved over time. Presently, rather than a gap persisting with regard to physical access to computers and the internet, it relates to a gap in the skills required to use the internet [46]. Moreover, the use of online public services is lower precisely among the section of society that needs them the most [47]. In the case of this research, which seeks to connect caregivers with volunteers, there is an asymmetry among caregivers, who, according to previous research, prefer to use a feature phone [12], and volunteers, who suggest a preference for a mobile application. Therefore, in order to communicate to groups on both sides of the digital divide, it is necessary to create and design technologies that are accessible and adapted to the needs of people with limited technical expertise [33], or to ensure the use of simple applications that are understood by both groups, such as WhatsApp.

Volunteers in this research would be willing to provide personal information for the purpose of volunteering. Specifically, the volunteer coordinators from the volunteering institution would manage and maintain volunteer information, including name, age, demographic information, availability and schedules, qualifications and skills, experience including previous volunteering, and overall levels of responsibility. They would also manage information related to volunteering beneficiaries, the location in which the volunteering will take place, and the duration of the help provided [33]. It should be noted that the use of social computing technologies requires that information belonging to members of the online community (or social network) is sufficiently visible for volunteering to take place and for other people to become motivated to participate, while remaining sufficiently private so that volunteers who prefer to do so are able to remain anonymous or manage their privacy preferences accordingly [39].

## 6. Conclusions

This study found that volunteers who provide respite care to the caregivers of bedridden older persons are willing to use technology to deliver volunteer care services. However, although they are willing to provide basic contact information, they are also aware of the digital skills gap that may exist between them and the caregivers, as well as being distrustful of unknown websites that provide information about potential care recipients. The presence of a volunteering institution that provides structure and is trustworthy is a critical factor for both volunteers and caregivers.

We would like to acknowledge our study limitations. First, this study was conducted in a specific context in Peñalolén, Chile, and to extrapolate our findings to a wider population we should consider these characteristics. Second, the participants of this study were potential volunteers, i.e., although most had volunteered before and were willing to do so again, they were not currently volunteering for this specific situation, so our results talk about their perceptions and expectations and not actual experience in this precise situation.

As future work, we will analyse volunteer and caregiver data, created manually by volunteering institutions, in order to design a system capable of connecting caregivers and volunteers.

## Appendix A

1. Have you ever voluntarily helped take care of someone? Who? What was your experience like?

2. Would you be willing to provide volunteer help to a dependent older adult, so that his/her caregiver can have some time off?

3. What type of help would you be willing to provide? How much time are you willing to provide for this help? How often would you be willing to provide this help?

4. Which characteristics should the recipient of care have?

5. What would help you gain trust to be able to voluntarily help a caregiver of a dependent older adult?

6. If a computer-based system was available to help people communicate to give and receive this type of help, what do you imagine it would be like? How would the system work? Which information would you like to access? Which information are you willing to provide?
